# The Abuse of Opium

**Published:** 1888-01

**Authors:** 


### THE ABUSE OF OPIUM.

Said the late Dr. Farrington, in his lecture upon Opium (*vide Clinical Materia Medica*, page 244): "No drug is more freely abused by both allopath and homœopath(!) than is the one we are studying to-day. I would I had both opportunity and ability to convince the practitioner of the old school of medicine of the absurdity of his indiscriminate use of opiates; and I could hope still more earnestly to dissuade homœopathicians from hiding their ignorance under the anodyne effects of an occasionally interpolated dose of Morphine or Laudanum. The one class, ignorant of any other means of assuaging pains, and the other class, too lazy to study their cases, seek relief for their patients in anodynes. Call them to task for their unscientific practice, and they meet you with the remark, 'My duty is to relieve the sick.' Let me rejoin: 'At any cost? Must you do what you know to be wrong?'" After a brief *résumé* of the *modus operandi* of Opium in its toxic action, Dr. Farrington says: "Now, gentlemen, let me ask, is it rational to assuage pain with a substance which paralyzes, and so relieves by taking away, *not the disease*, BUT THE ABILITY TO FEEL, THE CONSCIOUSNESS OF SUFFERING?"

